# Multicellular Ovarian Cancer Model for Evaluation of Nanovector Delivery in Ascites and Metastatic Environments

**DOI:** 10.3390/pharmaceutics13111891

**Published:** 2021-11-08

**Authors:** Stephen J. Winter, Hunter A. Miller, Jill M. Steinbach-Rankins

**Affiliations:** 1 School of Medicine, University of Louisville School of Medicine, Louisville, KY 40202, USA; stephen.winter.1@louisville.edu; 2 Department of Pharmacology and Toxicology, University of Louisville School of Medicine, Louisville, KY 40202, USA; hunter.miller@louisville.edu; 3 Department of Bioengineering, University of Louisville Speed School of Engineering, Louisville, KY 40202, USA; 4 Department of Microbiology and Immunology, University of Louisville School of Medicine, Louisville, KY 40202, USA; 5 Center for Predictive Medicine, University of Louisville, Louisville, KY 40202, USA

**Keywords:** ovarian cancer, spheroid model, nanoparticle transport

## Abstract

A novel multicellular model composed of epithelial ovarian cancer and fibroblast cells was developed as an in vitro platform to evaluate nanovector delivery and ultimately aid the development of targeted therapies. We hypothesized that the inclusion of peptide-based scaffold (PuraMatrix) in the spheroid matrix, to represent in vivo tumor microenvironment alterations along with metastatic site conditions, would enhance spheroid cell growth and migration and alter nanovector transport. The model was evaluated by comparing the growth and migration of ovarian cancer cells exposed to stromal cell activation and tissue hypoxia. Fibroblast activation was achieved via the TGF-β1 mediated pathway and tissue hypoxia via 3D spheroids incubated in hypoxia. Surface-modified nanovector transport was assessed via fluorescence and confocal microscopy. Consistent with previous in vivo observations in ascites and at distal metastases, spheroids exposed to activated stromal microenvironment were denser, more contractile and with more migratory cells than nonactivated counterparts. The hypoxic conditions resulted in negative radial spheroid growth over 5 d compared to a radial increase in normoxia. Nanovector penetration attenuated in PuraMatrix regardless of surface modification due to a denser environment. This platform may serve to evaluate nanovector transport based on ovarian ascites and metastatic environments, and longer term, it provide a means to evaluate nanotherapeutic efficacy.

## 1. Introduction

Ovarian cancer is a lethal gynecologic malignancy, with a 5 year survival rate for all stages and ethnicities of 49% [1,2]. Unlike other histological subtypes that yield higher survival rates, epithelial ovarian cancer (EOC) is the primary contributor to such dismal mortality. Often diagnosed late due to a lack of reliable biomarkers, EOC is typically characterized by its pattern of intraperitoneal invasion and dissemination and can be further categorized as high-grade serous (HGS) and nonserous (NS). HGS cells are typically derived from the epithelium of the fallopian tube fibria and represent a more aggressive and deleterious ovarian cancer, accounting for 70–80% of deaths from ovarian cancer alone. Conversely, NS are histologically linked to the endometrium and represent a more indolent form of disease progression [3,4,5].

Current treatment approaches for EOC primarily comprise surgery and chemotherapy, which often result in recurrence and meager therapeutic outcomes within 1–2 years of initial treatment. Major challenges facing ovarian cancer chemotherapy include toxic effects to healthy tissue, inadequate targeting, impaired transport through the tumor microenvironment (TME), and poor cellular internalization [6,7,8,9]. The systemic administration of chemotherapeutics contributes to these outcomes, as delivery is hindered by the very nature of advanced ovarian cancer, which includes poorly vascularized nodules that reside in the abdomen, liver, and lungs. As a result, inadequate therapeutic distribution and diffusion from systemic circulation lead to insubstantial drug concentrations in tumor tissue. In addition, multidrug resistance adversely impacts chemotherapeutic efficacy. New treatment approaches have focused on integrating more specific strategies, such as gene delivery and nanotherapy, with traditional anticancer agents to better target ovarian cancer, overcome multidrug resistance, and enhance therapeutic efficacy [10,11,12,13].

The epithelial-to-mesenchymal transformation (EMT) is a hallmark of invasive metastatic ovarian cancer, induced in the presence of a tumorigenic microenvironment. Cancer cells undergo EMT in the presence of transforming growth factor beta 1 (TGF-β1), inducing a phenotypic transformation from a differentiated adherent epithelial phenotype to a more motile mesenchymal phenotype that contributes to metastatic invasion [14,15]. Recent studies have shown that cellular interactions within the extracellular matrix (ECM) can lead to the reprogramming of the stromal environment and consequently an increase in ovarian cancer metastatic potential [15,16,17]. In ovarian cancer, the critical role of the TME, which consists of a complex arrangement of stromal cells (e.g., fibroblasts, macrophages, regulatory T-cells, myeloid-derived suppressor cells, endothelial cells, pericytes, and platelets); inflammatory cytokines; and extracellular matrix constituents (glycoproteins, proteoglycans, and polysaccharides) that communicate with the epithelial cancer cells and contribute to metastatic potential, is becoming increasingly recognized [18,19].

One of the primary contributors to EMT are cancer-associated fibroblasts (CAFs), which form heterotypic nodules with metastatic tumor cells [15,16,20]. Paracrine signaling from activated fibroblasts during the EMT process stimulates CAFs to engender a pre-metastatic niche in the peritoneum, ultimately leading to enhanced migration, nodule adhesion, and therapeutic resistance [21]. Additionally, normal fibroblasts that reside in the connective tissue of the peritoneum or ovary are transformed to a cancerous phenotype via a growth-factor-mediated pathway through paracrine signaling [22]. Recent studies [23] that seek to mimic these properties have shown that the fibroblast cell line medical research council cell strain 5 (MRC-5) can be chemically stimulated to an activated phenotype, leading to the initiation of EMT in ovarian cancer. This transformation can result in migration, cell cycle arrest, and resistance to apoptosis [24]. Moreover, MRC-5 cells have demonstrated compatibility with ascitic ovarian cancer cell lines such as SKOV-3 and have the ability to express tumorigenic properties when activated [16].

In addition to alterations observed within the tumor tissue environment, another hallmark feature of ovarian cancer is the induction of hypoxia, resulting from limited vascularization and correspondingly low levels of associated oxygen transport into tumor tissue. The initial damage from inefficient electron transport leads to the production of reactive oxygen species (ROS), resulting in reduced proliferation and apoptosis [25]. DNA damage repair mechanisms are upregulated in these harsh environments, ultimately resulting in adaptations and stabilization of the cell cycle [26,27]. Consequently, cancer cells may express a more aggressive phenotype that enables them to survive in a quiescent state and remain resistant to chemotherapeutic DNA damage. Specifically, in the ascites nodules, hypoxic regions develop in response to vascular depletion, hemostasis, and tissue diffusion limitations, impacting metabolic activity and cell proliferation [26,27].

In vitro models to study the metastatic progression of ovarian cancer have traditionally focused on monolayer cell cultures and tumor spheroid models. Although inheriting limitations of in vitro models, multicellular 3D tumor spheroid models express the characteristics of human tumors such as ECM distribution, cell composition, and pH, enabling a more accurate evaluation of preclinical therapeutic delivery and effectiveness relative to two-dimensional models [11]. Models based on tumor spheroid architectures have been used to study avascular ovarian tumor nodules [28,29], as the 3D tissue architecture provides similar environmental cues to simulate poorly vascularized tissue, including the generation of oxygen, nutrients, pH, and therapeutic diffusion gradients under controlled conditions [7,30]. Additionally, these spheroids have the ability to mimic the architecture of avascular nodules, which are representative of hypovascularized ovarian cancer metastases to the abdomen, liver and lungs. Recent studies have shown that micrometastatic nodules composed of ascitic-derived cancer cells superimposed on a CAF backbone are representative of the composition of ovarian cancer as it migrates to distant sites [20]. This finding highlights the importance of creating new models that accurately depict the TME and enable the exploration of targeted therapies that disrupt CAF influence on peritoneum implantation [20].

Previous work in our group has focused on the evaluation of nanoparticle (NP) transport in liquid overlay and hanging drop spheroids composed of a single-cell type and how various NP surface modifications might enable NPs to penetrate these solid tumors. Our team evaluated how “stealth” surface ligands such as polyethylene glycol (PEG) or cell-penetrating peptides such as MPG (unabbreviated notation) might alter the intratumoral distribution and intracellular uptake of these NPs in a monocellular spheroid system [31,32,33]. These studies provided the basis to explore how surface modifications might perform in a more representative multicellular ovarian cancer model.

Some of the complex biochemical and physiological features of ovarian tumorigenesis, their representation in spheroid models, and role in therapeutic resistance are highlighted in Table 1. A complex feature of ovarian cancer is that two distinct phases contribute to its metastatic and aggressive tumor phenotype. The early stage of ovarian cancer progression is characterized by cell detachment from primary ovarian tissue and migration to distant sites. This stage is dominated by a complex reorganization of the TME due to selective pressure from stromal activation and the hypoxic ascites environment. In the same way, unchecked cell proliferation leads to peritoneal invasion and the development of therapeutic resistance, which induces further alterations to the ECM architecture and embedding in the peritoneal wall. Epithelial cells in the ascites have a migratory and adherent phenotype that can leverage the invasive characteristics of the ECM and continue to enhance therapeutic resistance [34,35,36].

In light of these complex features, the goal of this study was to develop an in vitro ovarian cancer model that represents EOC in both the ascites and at its site of invasion to provide a more representative platform in which to study and target not only malignant epithelial cells but also components of the tumorigenic microenvironment. We sought to develop a multicellular spheroid model composed of human ovarian ascites adenocarcinoma and fibroblast cells to better represent the stromal cell interactions and hypoxic conditions present in ovarian cancer. Tumor spheroids were embedded within a polypeptide scaffold to investigate the impact of this new tumor microenvironment, along with oxygenation and cell activation, on spheroid growth [26,37]. Additionally, we sought to determine the impact of these more complex features on NP transport, relative to our previous observations in a less complex hanging drop model [31,32,33]. We hypothesized that alterations to the TME along with inclusion of a peptide-based scaffold, PMX, to represent the site of metastasis, would lead to enhanced cell growth, migration, and altered NP transport, which may, in future work, help to inform delivery vehicle design for the challenging microenvironment of clinical ovarian cancer. We evaluated how spheroid coculture conditions impact ovarian tumor growth and migration in different surrounding environments that more accurately represent features such as cell activation and hypoxia, signatures of ascites to peritoneal transitions. Using a peptide-based ECM and traditional hanging drop model, we then compared NP transport within these different 3D environments and evaluated the effect of NP modification on spheroid penetration, importantly identifying how these differences may provide insight into nanotherapy within a more representative microenvironment.

**Table 1:** Epithelial ovarian cancer (EOC) biphasic model. Description of the complex biochemical and physiologic changes involved in ovarian tumorigenesis, leading to resistance, and their representations in in vitro spheroid models. Ovarian cancer progression can be divided into two key stages based on tumor cell metastasis to the ascites and peritoneum. During both ascites and peritoneal invasion, the physiological features and their relevance to the pathophysiology of ovarian cancer metastasis were recapitulated in these in vitro models. An increase or decrease in tumor characteristics is denoted by “↑” and “↓”, respectively, while “➣” indicates a transition or induction of a given characteristic.

Epithelial Ovarian Cancer (EOC) Model				
Life-Cycle Ovarian Cancer	Physiological Features	Origin	Reference	Model
Ascites	primary tumor cells and CAFs organize into dense heterotypic spheroids	EMT ➣ EOC cells w/↓ self-adhesion	[16,24,38]	activated MRC-5s w/TGF-β1 cocultured with SKOV-3 cells without ECM mimetic
	↑ spheroid density	activated TME ➣ CAF phenotype ➣ ↑ stress, ↑ contractility, ↑ alignment of ECM	[39,40,41,42,43,44,45]	activated TME ➣ ↓ change in spheroid radius
	↓ cell proliferation and apoptosis in peripheral zone	low pH ➣ hypoxia ➣ ROS ➣ DNA damage	[16,22,25,38,46]	hypoxia ➣ ↓ change in spheroid radius and ↑ blebbing of cell membranes
	↓ particle transport/penetration into spheroid	activated TME ➣ CAF phenotype ➣ ↑ stress ↑ contractility, ↑ alignment of ECM	[39,40,41,42,43,44,45,47,48,49]	↓ NP penetration and cellular uptake
	↑ therapeutic resistance	DNA damage ➣ DNA repair mech ➣ EOC resistant to apoptosis ➣ aggressive phenotype	[7,20,24,27,38,50,51]	Future work: IC-50 w/chemotherapeutic
Peritoneal Migration	heterotypic spheroids in ascites adhere to peritoneum	EMT + activated TME ➣ invasive EOC phenotype + invasive ECM	[27,38,52]	activated MRC-5s w/TGF-β1 cocultured with SKOV-3 cells w/PMX ECM mimetic
	↑ migratory behavior of EOC	EMT + activated TME ➣ invasive EOC phenotype + invasive ECM	[39,40,41,42,43,44,45,51]	↑ change in spheroid radius
	↓ particle transport/penetration into spheroid	activated TME ➣ CAF phenotype ➣ ↑ stress, ↑ contractility, ↑ alignment of ECM	[39,40,41,42,43,44,45,47]	↓ NP penetration and cellular uptake
	↑ therapeutic resistance	DNA damage ➣ DNA repair mech ➣ EOC resistant to apoptosis ➣ aggressive phenotype	[7,20,24,27,38,50,51]	Future work: IC-50 w/chemotherapeutic

## 2. Materials and Methods

### 2.1. Cell Lines

SKOV-3 human ovarian ascites adenocarcinoma cells (ATCC^®®^ HTB-77) and MRC-5 human fetal normal lung fibroblast cells (ATCC^®®^ CCL-171) were obtained from the American type culture collection (ATCC). The SKOV-3 cell line was selected for its aggressive phenotype and ability to form micronodules with a CAF proxy in vitro such as MRC-5 and arguably represents ascites and migratory phases of ovarian cancer metastases. SKOV-3 was derived from the ascites of a serous cystadenocarcinoma and shares both biomarkers of HGS and HS histotypes, although sometimes categorized as NS. Both cell lines were cultured in Dulbecco’s modified Eagle’s medium/nutrient mixture F-12 (DMEM/F-12, Life Technologies) supplemented with 10% fetal bovine serum (FBS, Invitrogen) and 1% streptomycin, at 20% O_2_, 5% CO_2_ and 37 °C. Immortalized SKOV-3 cells were used between passage numbers 15 and 40, while MRC-5 cells, were used between passage numbers 6 and 16.

### 2.2. Activation of Fibroblasts to Tumorigenic Phenotype

A subset of MRC-5 cells was activated with TGF-β1 to assess the effect of activation on cell migration within the tumor microenvironment. MRC-5 cells were cultured in a T75 flask until reaching 70–80% confluence and were subsequently washed one time with Dulbecco’s phosphate buffered saline (DPBS, 1×) to remove latent serum-derived TGF-β1 from the media. Cells were then incubated in serum-free media (DMEM/F12) for 48 h prior to activation, to stimulate entry to the G_0_ quiescent stage. After 48 h, serum-free media were removed, and MRC-5 cells were subsequently transformed to an activated phenotype by incubating with 20 ng/mL TGF-β1 for an additional 48 h [16,25,53,54].

### 2.3. Hanging Drop Multicellular Tumor Spheroid Growth

**Figure 1:**
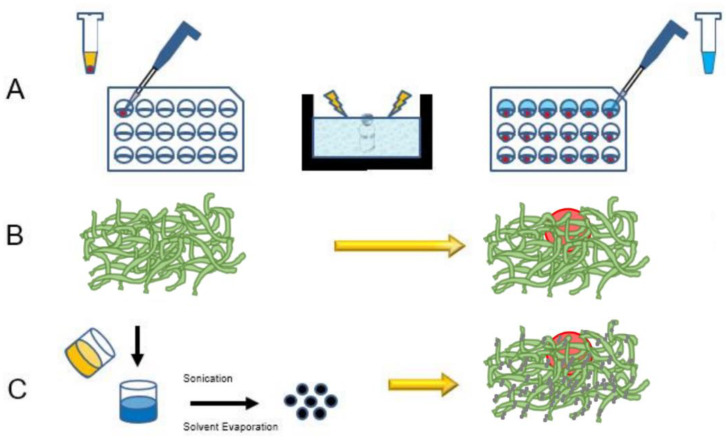
Schematic of experimental procedure. (**A**) Spheroids (shown in red) were embedded within PMX to better represent the tumor microenvironment and extracellular matrix (PMX solution shown in blue). (**B**) PMX self-assembles into a nanofiber architecture (green) around the tumor spheroid after exposure to media salts. (**C**) Spheroids embedded in PMX were treated with two NP formulations PEG and MPG (shown in yellow/navy), and the distribution was evaluated after 24 h incubation in normoxic and hypoxic conditions.

An overview of the spheroid embedding process and PMX assembly is shown in Figure 1. To form hanging drop spheroids, ultralow attachment plates (#4515, Corning, Corning, NY, USA) were utilized. Nonactivated MRC-5, activated MRC-5, and SKOV-3 cells were incubated with DMEM/F-12 media in a T75 flask until reaching 70–80% confluence. For all cell cultures, media were removed from each T75 flask, and cells were trypsinized. Cells were subsequently seeded for 5 days in 100 μL of DMEM/F12 at a 1:1 SKOV-3: MRC-5 cell ratio, with 5000 total cells per well in a 96-well plate (Figure 1A). Either nonactivated (SKOV-3/MRC-5) or activated (SKOV-3/MRC-5(A)) cells were cocultured to assess the impact of MRC-5 activation on cell proliferation and migration.

### 2.4. Hypoxic Incubation

For cell groups that were evaluated under hypoxic conditions, spheroids were incubated in 5% oxygen and 5% CO_2_ during the spheroid formation and growth stages.

### 2.5. Addition of Polypeptide Scaffold to Multicellular Tumor Spheroids

A subset of spheroids was embedded within PuraMatrix (PMX, Corning, 354250, Corning, NY, USA) hydrogel scaffolds to evaluate invasion and, in later experiments, nanoparticle transport [55,56] (Figure 1B,C). After two days of hanging drop growth, spheroids were introduced to PMX to minimize disruption of spheroid integrity. The PMX stock solution (10 mg/mL) was sonicated in a water bath for 30 min to reduce viscosity and was subsequently centrifuged for 10 min at 2400 rcf to eliminate bubbles. Stock solution was diluted to 2.5 mg/mL in 10% sucrose. Half of the spheroid culture media (50 μL) was removed from each well of the 96-well plate and replaced with 2.5 mg/mL PMX solution. Due to the acidic pH of PMX, 33 μL of the new PMX/media was removed after 30 min to minimize spheroid disruption and replaced with 50 μL fresh media [57].

### 2.6. Characterization of Spheroid Growth

Radial growth of multicellular ovarian spheroids was measured in activated and hypoxic conditions over 5 days. A model of spheroid invasion into distal tissue of the peritoneum was further evaluated by measuring the outward radial growth of SKOV3 cells in the ECM mimetic, PMX. Spheroid invasion into PMX was characterized for all groups (SKOV-3, MRC-5, MRC-5(A), SKOV-3/MRC-5, and SKOV-3/MRC-5(A)) over 3 days after being cultured in PMX for 5 days. PMX-embedded spheroids were allowed to incubate for 24 h before representative images were taken. Each day, spheroids were imaged with an epifluorescent microscope (Axiovision 4, Zeiss, White Plains, NY, USA) under transmitted light using a 10× objective with phase contrast. For images that exceeded the optical field of view, digital mosaics were made by scanning the well and constructing a 2 × 2 stitched matrix of spheroid images. A minimum of three representative samples per spheroid group were analyzed using ImageJ, and 2D radial invasion was measured by selecting the ROI (region of interest) and utilizing the measurement tool to quantify change in maximal cross-sectional radii over 3 days. An assumption of a spherical surface area was not appropriate for PMX-cultured spheroids, due to their nonspherical irregularity; therefore, the maximum cross-sectional radii were measured by taking the average of 8 lines drawn from the edge of the periphery to the center of each spheroid.

### 2.7. Nanoparticle Synthesis 

PLGA NPs encapsulating the fluorophore coumarin 6 (C6) were synthesized as previously described [31,58] to visualize NP distribution within the tumor spheroids via fluorescence microscopy. Carboxyl-terminated poly(lactic *co*-glycolic acid, PLGA) (0.55–0.75 dL/g, LACTEL^®®^) was used to synthesize 100–200 mg C6 NP batches using an oil-in-water (o/w) single-emulsion technique [31,58,59]. In brief, C6 was dissolved in methylene chloride (DCM) overnight at a concentration of 15 μg C6 per mg of PLGA. The following day, the PLGA/C6/DCM solution was added dropwise to a 5% polyvinyl alcohol (PVA) solution of equal volume, vortexed, and sonicated. The resulting NPs were hardened in 0.3% PVA during solvent evaporation for 3 h [31,32,33]. After hardening, NPs were centrifuged and washed three times at 4 °C, in deionized water (diH_2_O) to remove residual solvent.

For surface-modified NPs, a slightly adapted protocol was used. Avidin-palmitate for surface conjugation was synthesized as previously described [31,32,33,58,60]. Forty milligrams of avidin (A9275, Sigma, St. Louis, MO, USA) were dissolved in 4.8 mL of 2% sodium deoxycholate (NaDC) in phosphate buffered saline (PBS) warmed to 37 °C. Palmitic acid-NHS (PA-NHS, Sigma, St. Louis, MO, USA) was dissolved in 2% NaDC to a final concentration of 1 mg/mL and sonicated until well-mixed. The PA-NHS solution (3.2 mL) was added dropwise to 4.8 mL of the avidin NaDC solution and reacted overnight at 37 °C. The following day, the reaction was dialyzed in 1200 mL of 0.15% NaDC in PBS heated to 37 °C. Free PA-NHS was dialyzed overnight at 37 °C using 3500 MWCO tubing to remove free palmitic acid. After overnight dialysis, the dialysis tubing contents were transferred to a storage vial and stored at 4 °C until use.

Surface-modified NPs were synthesized similarly to unmodified NPs with the addition of avidin-palmitate (1 mg/mL) to the 5% PVA solution during the first emulsion, as previously described [31,32,33]. NPs were washed after hardening and centrifuged at 4 °C, twice in deionized water (diH_2_O) to remove residual solvent. Avidin-modified NPs were collected after the first wash and incubated for 30 min with biotinylated ligands, MPG (unabbreviated notation, 3177 Da, GenScript, Piscataway, NJ, USA), and polyethylene glycol (PEG, 5000 Da, Nanocs, Inc., ThermoFisher, Waltham, MA, USA), at a molar ratio of 3:1 ligand:avidin in PBS. Surface modification with PEG has been shown to enhance systemic and interstitial circulation times as well as to improve penetration through the tumor interstitium due to its hydrophilic properties [31,32,33]. Surface modification with MPG has been shown to dramatically increase cellular internalization due to its cationic and lipophilic properties [31,32,33]. After surface conjugation, the NPs were washed with diH_2_O and centrifuged twice, frozen, and lyophilized. All NPs were stored at −20 °C after synthesis.

### 2.8. Nanoparticle Characterization

The physical properties of the NP were confirmed using scanning electron microscopy (SEM, Zeiss SUPRA 35VP, White Plains, NY, US) to verify NP morphology. Dry NPs were mounted on carbon tape and sputter-coated with a thin layer of gold (25 nm) under vacuum in an argon atmosphere for 30 s (Dynavac Mini Coater, Dynavac, Hingham, MA, USA. Average particle diameter and size distribution were determined from SEM images of at least 400 particles per batch using image analysis software (ImageJ, National Institutes of Health) [32,33]. Zeta potential and dynamic light scattering were measured with a Zetasizer Nano ZS (Malvern) in diH_2_O to determine particle charge and hydrated diameter [32,33].

### 2.9. Nanoparticle Distribution 

To assess NP distribution in tumor spheroids as a function of surface modification, both MPG- and PEG-modified NP formulations were evaluated. Unembedded spheroids (non-PMX) and spheroids embedded within PMX were both cultured for 5 days and subsequently incubated with 50 μg/mL NPs for 24 h (Figure 1C). After treatment, both non-PMX and PMX spheroids were transferred to Eppendorf tubes, washed with 0.2 mL of 1 × PBS, and fixed with 0.2 mL of 4% paraformaldehyde. Spheroids were subsequently permeabilized with 0.2 mL of 1% Triton-X, washed twice with 0.2 mL PBS, and stained with 0.2 mL of 4 μg/mL Hoechst in 1 × PBS. Spheroids were incubated for fixation, permeabilization, and nuclear staining for 10 or 20 min each, for non-PMX and PMX spheroids, respectively. Finally, spheroids were washed with 0.2 mL of PBS and once in 0.2 mL of DI water, suspended in 50 μL PBS, and immediately transferred to imaging dishes (P35G-0-14-C, MatTek, Ashland, MA, USA).

Nanoparticle uptake and distribution within the spheroids were assessed via confocal microscopy (LSM 710, Zeiss, White Plains, NY, USA), and image analysis was performed using Zeiss ZEN 2011 software package. The following laser settings: 4′6-diamidino (DAPI) and GFP were used to visualize Hoechst (blue, cell nuclei) and C6 (green, within NPs), respectively. A laser intensity of 2 and a gain of 600 were used for the DAPI/Hoechst channel, while a laser intensity of 2 and a gain of 600 were maintained for the GFP/C6 channel across experiments. The Zeiss ZEN 2011 software package was utilized to generate average intensity projections (AIPs) from the composite z-stacks of the tumor spheroids. At least 3 representative samples were taken from each treatment group, and cross-sectional images into the projection plane of the AIP were created. NP penetration was quantified by plotting the mean fluorescence intensity (MFI) for each optical reconstruction of the cross-section of which the averages and standard deviations are reported. NP internalization was then assessed by analyzing the area under the curve (AUC, MFI-μm) of the generated distribution profiles using a trapezoidal approximation in Excel.

### 2.10. Statistical Analysis

All experiments were conducted with a minimum sample size of n = 3. Data were analyzed using a one-way ANOVA with a *p* value of 0.05 or less defined as statistically significant. For time-independent comparisons, percentage difference was calculated, as in Equation (1). For time-dependent comparisons, percent change was calculated, as in Equation (2).

*abs* (X_1 − X_2)/((X_1 + X_2)/2) = Percentage Difference (1)

(X_final − X_initial)/X_initial = Percent Change(2)

## 3. Results

### 3.1. Non-PMX Spheroid Growth as a Function of Cell Activation and Oxygenation

#### 3.1.1. Impact of Cell Activation

**Figure 2:**
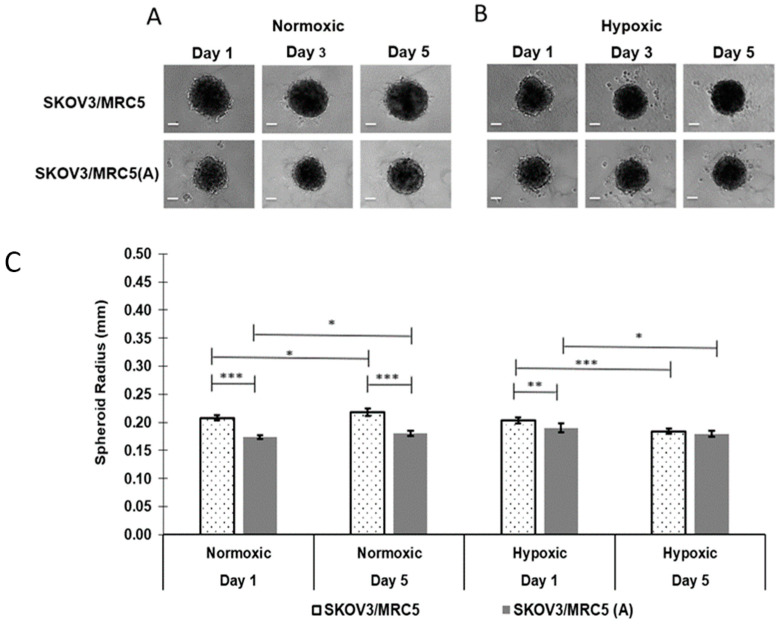
Non-PMX spheroid growth patterns as a function of activation and oxygenation over 5 days measured as maximum cross-sectional radius. Representative phase-contrast images of (**A**) normoxic and (**B**) hypoxic non-PMX nonactivated (SKOV-3/MRC-5) and activated (SKOV-3/MRC-5(A)) spheroid growth. (**C**) While spheroids increased in size in normoxia, they decreased in size in hypoxia, with stromal activation overall yielding smaller volumes. Data were deemed statistically significant using one-way ANOVA (* *p* ≤ 0.05, ** *p* ≤ 0.005, *** *p* ≤ 0.0005). Error bars represent the mean ± standard deviation. Scale bars represent 100 μm.

To gain insight regarding the behavior of ovarian tumor growth, SKOV-3/MRC-5 spheroid growth and migration were observed without or after incorporation within an ECM mimetic (PMX) that was intended to recapitulate some features of tumor implantation and ascites sites. To establish a baseline, the impact of cell activation and environmental coculture conditions on the radial growth of nonactivated and activated SKOV-3/MRC-5 spheroids was measured in both normoxic and hypoxic conditions in the absence of PMX. Representative images of both nonactivated and activated SKOV-3/MRC-5 spheroids cocultured without PMX in normoxic (Figure 2A) and hypoxic conditions (Figure 2B) are shown after 1, 3, and 5 days of growth. Differences in maximum spheroid radii, as a function of activation state, oxygen level, and growth duration are quantified in Figure 2C. The percentage difference (Equation (1)) and percent change (Equation (2)) equations were used to compare time-independent and time-dependent changes, respectively.

Under normoxic conditions, nonactivated non-PMX spheroids were 18.0% (0.208 ± 0.004 mm vs. 0.174 ± 0.003 mm, *p* ≤ 0.0005) and 19.1% (0.218 ± 0.007 mm vs. 0.180 ± 0.004 mm, *p* ≤ 0.0005) larger, as defined by the maximum cross-sectional radius, relative to activated non-PMX spheroids after 1 and 5 days in culture, respectively (Figure 2C). Similarly, nonactivated non-PMX spheroids cultured in hypoxic conditions were 6.9% larger, relative to activated non-PMX spheroids after 1 day in culture (0.204 ± 0.004 mm vs. 0.190 ± 0.006 mm, *p* ≤ 0.005). However, no statistical significance was observed between nonactivated and activated non-PMX spheroids cultured in hypoxic conditions after 5 days of growth (0.184 ± 0.004 mm vs. 0.179 ± 0.005 mm, *p* > 0.05). Overall, in both normoxic and hypoxic conditions, spheroids composed of nonactivated fibroblasts were observed to have larger radii at days 1 and 5 of growth, relative to activated spheroids.

#### 3.1.2. Impact of Normoxic vs. Hypoxic Environments on Spheroid Size over 5 Days

In addition to the effect observed from cell activation, both nonactivated and activated non-PMX spheroids cultured in normoxic conditions, respectively, experienced a 4.9% (0.208 ± 0.004 mm to 0.218 ± 0.007 mm, *p* ≤ 0.05) and 3.7% (0.174 ± 0.003 to 0.180 ± 0.005 mm, *p* ≤ 0.05) increase in maximum cross-sectional radii over 5 days of growth (Figure 2C). By contrast, both nonactivated and activated cells cultured in hypoxic conditions, respectively, showed a 9.6% (0.204 ± 0.004 mm to 0.184 ± 0.004 mm, *p* ≤ 0.0005) and 5.7% (0.190 ± 0.006 mm to 0.179 ± 0.005 mm, *p* ≤ 0.05) decrease in maximum cross-sectional radii after 5 days of growth (Figure 2C). These results indicate that while in normoxia, the spheroid size increase was comparable between activated and nonactivated cells, in hypoxia, the spheroid regression was attenuated in an activated state. Overall, both nonactivated and activated spheroids cultured in normoxic conditions were larger, less diffuse, and more regular in morphology than those cultured in hypoxic conditions (Figure 2A,B).

### 3.2. Spheroid Growth as a Function of PMX Incorporation 

Previous work in our group has focused on evaluating tumor spheroid formation in the hanging drop model, which consists of forming 3D cellular architectures driven by gravity. While the hanging drop model may be used to form unicellular or multicellular spheroids, the surrounding extracellular matrix lacks a consistent and reproducible composition and fails to adequately incorporate cell adhesion dynamics responsible for the metastatic characteristics of EOC [16,61]. Herein, we sought to implement a more advanced tumor spheroid formation approach that incorporates the gravitational design of the hanging drop model with a more representative matrix for ovarian cancer cells. Multicellular spheroids were formed and then introduced to PMX, a physiological scaffold that has been shown to enhance cell migration relative to hanging drop alone, to assess its impact on spheroid growth and migration.

**Figure 3:**
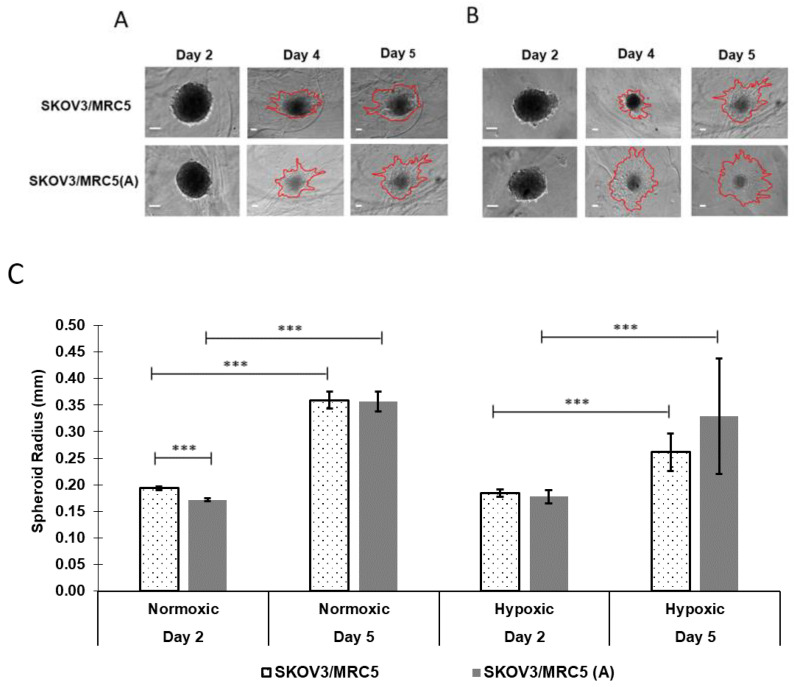
Invasion of spheroids in PMX as a function of activation and oxygenation over 5 days measured as maximum cross-sectional spheroid radii. Representative phase-contrast images of (**A**) normoxic and (**B**) hypoxic PMX nonactivated (SKOV-3/MRC-5) and activated (SKOV-3/MRC-5(A)) spheroid growth. (**C**) Spheroids increased in size in both normoxia and hypoxia, with no difference between the activated and nonactivated stromal conditions by 5 days. Data were deemed statistically significant using one-way ANOVA (*** *p* ≤ 0.0005). Error bars represent the mean ± standard deviation. Scale bars represent 100 μm.

Representative images are shown for nonactivated and activated SKOV-3/MRC-5 spheroids cocultured in normoxic and hypoxic PMX hydrogels after 2, 4, and 5 days (Figure 3A,B). Observational time points differed from those in non-PMX spheroids to allow 24 additional hours for cells to develop a 3-dimensional architecture to withstand PMX introduction. Spheroids incorporated in PMX (Figure 3A,B) exhibited a more diffuse morphology relative to the spherical morphology of the hanging drop non-PMX spheroids shown in Figure 2A,B. Red lines delineate the leading edge of the spheroid as it expands into surrounding PMX. Control experiments conducted using MRC-5 only spheroids and SKOV-3 only spheroids showed that no appreciable growth or migration was observed in MRC-5-only spheroids, suggesting that SKOV-3 cells are primarily responsible for the increased proliferation and migration. Overall, spheroids cultured in PMX attained larger sizes relative to non-PMX spheroids. After five days of growth, both nonactivated and activated spheroids cultured in PMX and normoxic conditions were 48.7% (0.218 ± 0.007 mm vs. 0.359 ± 0.016 mm, *p* ≤ 0.0005) and 65.6% (0.180 ± 0.005 mm vs. 0.356 ± 0.019 mm, *p* ≤ 0.0005) larger in radii (Figure 3C), relative to the corresponding hanging-drop non-PMX spheroids Figure 2C), respectively. In hypoxic conditions, nonactivated and activated spheroids in PMX (Figure 3C) were 34.7% (0.184 ± 0.004 mm vs. 0.261 ± 0.035 mm *p* ≤ 0.0005) and 59% (0.179 ± 0.005 mm vs. 0.329 ± 0.109 mm *p* ≤ 0.05) larger in radii, respectively, relative to the corresponding hanging-drop spheroids (Figure 2C) after 5 days, demonstrating the impact of PMX to promote cell migration.

### 3.3. PMX Spheroid Growth as a Function of Cell Activation and Oxygenation

In addition to assessing cell migration based on non-PMX relative to PMX inclusion, cell migration was evaluated in PMX as a function of cell activation and spheroid oxygenation. Spheroid migration into the surrounding PMX was quantified by measuring the maximum cross-sectional spheroid radii at days 2, 4, and 5 (Figure 3C).

#### 3.3.1. Impact of Cell Activation

Under normoxic conditions, nonactivated PMX spheroids were 12% (0.194 vs. 0.172 mm, *p* ≤ 0.0005) larger, as defined by the maximum cross-sectional radius, relative to activated PMX spheroids after 2 days in culture (Figure 3C). No statistical significance was observed between nonactivated vs. activated PMX spheroids cultured in normoxic conditions after 5 days of growth (0.359 ± 0.016 mm vs. 0.356 ± 0.019 mm, *p* > 0.05). Similarly, no statistical significance was observed between nonactivated vs. activated PMX spheroids cultured in hypoxic conditions after 2 days (0.185 ± 0.007 mm vs. 0.177 ± 0.012 mm, *p* > 0.05) and 5 days (0.261 ± 0.035 mm vs. 0.329 ± 0.109 mm, *p* > 0.05). Of these groups, the only difference observed based on cell activation was in a normoxic environment, in the very early stages of growth (day 2).

#### 3.3.2. Impact of Normoxic vs. Hypoxic Environments on Tumor Size over 5 Days

Both nonactivated and activated spheroids cultured in PMX, under normoxic conditions for 2–5 days, demonstrated increases in maximal cross-sectional radii of 85.4% (0.194 ± 0.003 mm to 0.359 ± 0.016 mm, *p* ≤ 0.0005) and 107.4% (0.172 ± 0.003 mm to 0.356 ± 0.019 mm, *p* ≤ 0.0005), respectively, while similar spheroids cultured in hypoxic conditions increased by 41.4% (0.185 ± 0.007 mm to 0.261 ± 0.035 mm, *p* ≤ 0.0005) and 85.5% (0.177 ± 0.012 mm to 0.329 ± 0.109 mm, *p* ≤ 0.0005) during the same time frame (Figure 3C). In comparison, non-PMX spheroid cultures in similar conditions saw comparatively diminished changes (4.9%, 3.7%, −9.6%, and −5.7%) in maximal cross-sectional radii over 5 days (Figure 2C). These data indicate that the incorporation of cells in PMX, significantly increased spheroid growth potential, relative to non-PMX conditions and that cells cultured in PMX under normoxic conditions experienced increased growth relative to PMX-cultured cells in hypoxic conditions. Furthermore, in both normoxic and hypoxic environments, activated cells experienced increased relative growth over 5 days, relative to their nonactivated counterparts. These data indicate that both oxygenation and fibroblast activation contribute to the migratory behavior of SKOV-3/MRC-5(A) spheroids.

### 3.4. Nanoparticle Penetration into Multicellular Tumor Spheroids in Non-PMX and PMX Environments

To provide further insight into how these models may be used to study nanovector delivery, NP transport was characterized within non-PMX and PMX cultured spheroids.

#### 3.4.1. NP Transport in Non-PMX Spheroids

SKOV-3/MRC-5 and SKOV-3/MRC-5(A) cocultures were treated with NPs that were modified with either a stealth (PEG) ligand or a cell-penetrating peptide (MPG) to assess how PMX incorporation, oxygen level, and fibroblast activation impacted NP transport. Previous work by our group and others [47,58] has demonstrated how “stealth” surface ligands such as PEG and other ligands, might alter the intratumoral distribution of NPs in a monocellular spheroid system [31,32,33]. By contrast, cell-penetrating peptides, such as MPG, that have cationic and often lipophilic characteristics, have demonstrated increased cellular internalization, which is important for therapeutic effect. These studies provided the basis to explore how these surface modifications might perform in a more representative multicellular ovarian cancer model.

In brief, NP size and morphology were previously confirmed using SEM imaging and ImageJ processing. Unhydrated NPs demonstrated a spherical morphology, with diameters measuring 160–180 nm [31]. Hydrated NP surface charges were measured using a Zetasizer (Malvern). Unmodified NPs had a negative surface charge of −26.6 ± 1.1 mV; while PEG- and MPG-modified NPs measured −22.0 ± 1.4 and −8.5 ± 0.4 mV, respectively, validating surface ligand conjugation [31].

**Figure 4:**
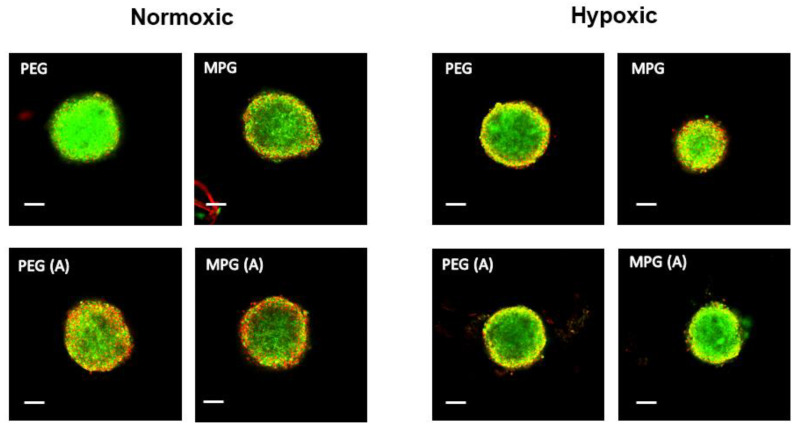
Representative fluorescence images of non-PMX spheroids including nonactivated and activated cells cultured in both normoxic and hypoxic conditions. Spheroids were incubated with 50 μg/mL NPs for 24 h after 5 days in culture. Green channel represents coumarin 6 NPs while red represents DAPI stained cells. Scale bars represent 100 μm.

**Figure 5:**
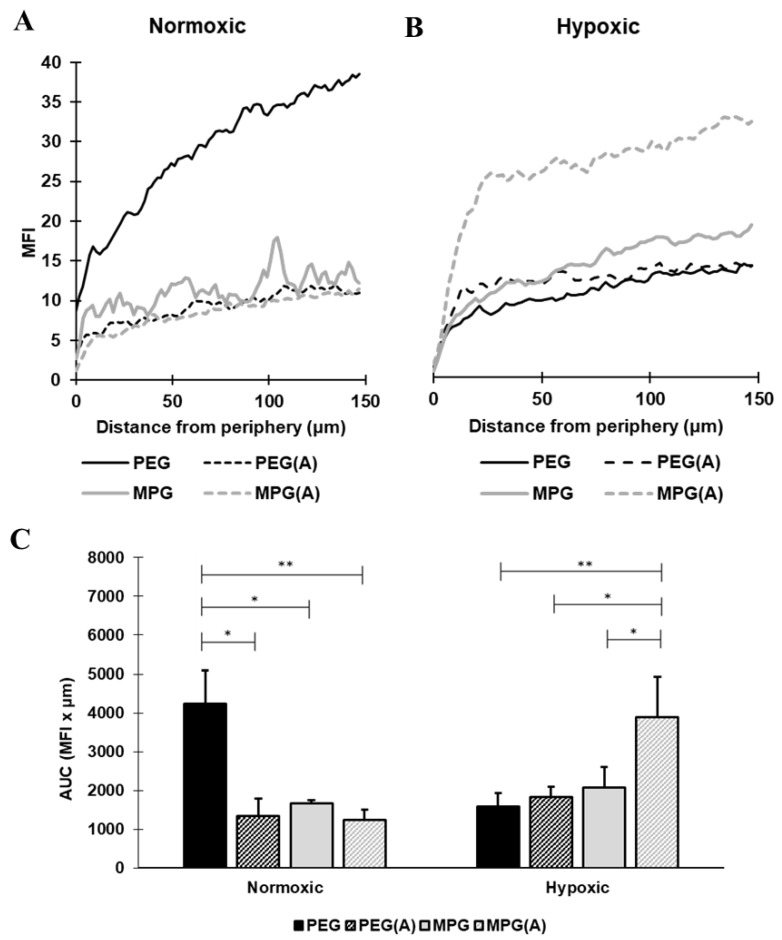
PEG NPs penetrated most deeply into spheroids composed of nonactivated SKOV-3/MRC-5 cells in normoxic conditions, while MPG NPs penetrated most deeply into spheroids composed of activated SKOV-3/MRC-5 cells in hypoxic conditions. (**A**,**B**) Quantification of surface-modified PLGA NP transport into the tumor center as a function of MFI in normoxic and hypoxic conditions. (**C**) Quantification of area under the MFI curves. Data were deemed statistically significant using a one-way ANOVA (* *p* ≤ 0.05, ** *p* ≤ 0.005). Error bars represent the mean ± standard deviation.

Representative confocal microscopy images of hanging drop non-PMX spheroids as a function of NP modification type are shown as a single slice at the midpoint of the *z*-axis, and representative average intensity composites (AICs) are provided for each z-stack (Figure 4, Supplementary Materials Figure S1). NP penetration as a function of surface modification is shown as the MFI versus distance from the non-PMX spheroid periphery in Figure 5A,B, while Figure 5C depicts NP concentration in spheroids as the AUC (MFI × µm). Overall, for both PEG and MPG NPs in nonactivated and activated spheroids, NP concentration increased from the spheroid periphery toward the center of spheroid mass.

For spheroids cultured in normoxic conditions (Figure 4 and Figure 5A,C), PEG-modified NPs administered to nonactivated spheroids penetrated most deeply, relative to MPG NPs in nonactivated spheroids (*p* ≤ 0.05) and both PEG and MPG NPs in activated spheroids (PEG(A), MPG(A)) (*p* ≤ 0.05). Within hypoxic spheroids (Figure 5B,C), MPG NPs penetrated most deeply into activated spheroids (MPG(A)), relative to MPG in nonactivated spheroids (*p* ≤ 0.05), and PEG NPs in activated and nonactivated spheroids (*p* ≤ 0.05, *p* ≤ 0.005). As a function of oxygen level in nonactivated tumor spheroids, PEG NPs in a normoxic environment had a significantly increased distribution relative to PEG NPs in a hypoxic environment. By contrast, PEG NPs in activated spheroids had similar transport properties in normoxic and hypoxic environments, whereas MPG(A) (hypoxic) had a significantly higher uptake than MPG(A) in normoxic conditions. In summary, PEG NPs penetrated most deeply into nonactivated SKOV-3/MRC-5 normoxic spheroids, while MPG NPs penetrated most deeply into activated hypoxic spheroids. The penetration of other groups into normoxic and hypoxic environments was similar (*p* > 0.05).

#### 3.4.2. NP Transport in PMX Spheroids

**Figure 6:**
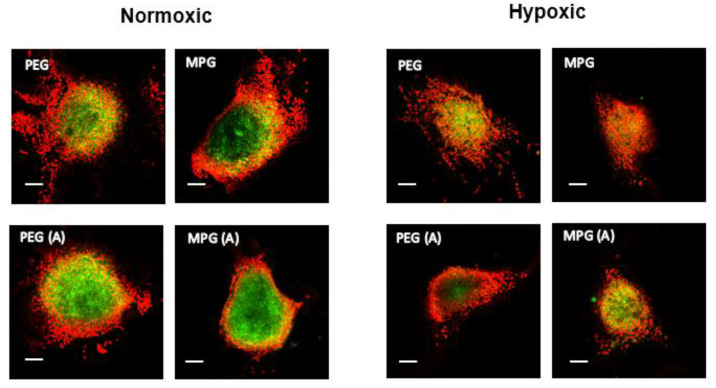
Representative fluorescence images of PMX spheroids including nonactivated and activated cells cultured in both normoxic and hypoxic conditions. Spheroids were incubated with 50 μg/mL NPs for 24 h after 5 days in culture. Green channel represents coumarin 6 NPs, while red represents DAPI stained cells. Scale bars represent 100 μm.

**Figure 7:**
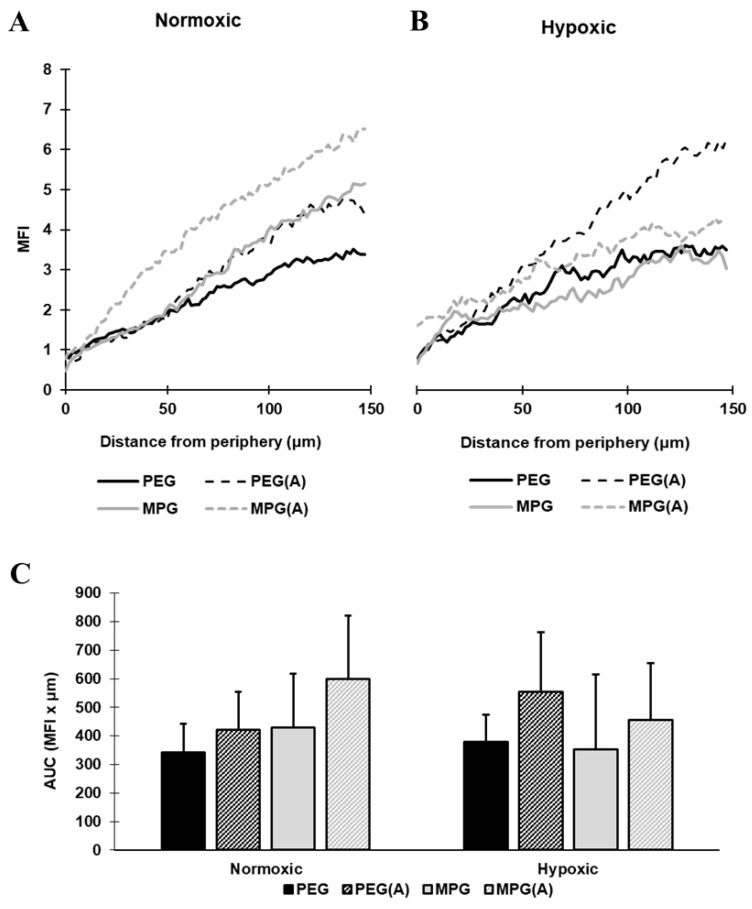
No statistical significance was observed between PEG and MPG NP penetration into PMX spheroid composed of activated SKOV-3/MRC-5 cells cultured in normoxic or hypoxic conditions. (**A**,**B**) Quantification of surface-modified PLGA NP transport into the tumor center as a function of MFI in normoxic and hypoxic conditions. (**C**) Quantification of area under the MFI curves. No statistical significance was observed as a function of activation, hypoxia, or NP type. Error bars represent the mean ± standard deviation.

NP penetration through PMX-embedded spheroids is shown in representative confocal microscopy images (Figure 6) as a function of NP modification type (PEG, MPG), while Supplementary Materials Figure S2 shows representative AICs of the z-stack images. Figure 7A,B show the MFI with respect to distance from spheroid periphery, and Figure 7C shows the AUC. In this invasion-permissive PMX model, PEG NPs appear to outperform MPG; however, no statistically significant difference was established.

Overall, no statistical significance was observed in NP penetration between: nonactivated vs. activated spheroids, PEG vs. MPG NPs, and hypoxic vs. normoxic conditions in PMX spheroids. However, a significant decrease in NP penetration was observed in PMX-embedded spheroids (Figure 7) relative to non-PMX spheroids (Figure 5), as shown by the quantification of area under the normalized MFI curves. Although it is likely that NP transport was impacted by oxygenation conditions, we were unable to observe significant changes relative to the more dominant effect of PMX.

## 4. Discussion

An increasing demand for in vitro models that can recapitulate features of the tumor microenvironment has provided an impetus to develop cocultures that incorporate tumor microenvironment constituents and stromal cell interactions [18]. Multicellular spheroid models, developed for other cancer types, have focused on optimizing extracellular matrix composition and architecture, incorporated cell types, and associated culture conditions [11,62]. Recent work has begun to explore ovarian cancer-specific models by integrating relevant cell types and cytokines within a scaffold that more accurately models the native ECM. Very recently, heterotypic spheroid models, composed of fibroblasts and epithelial ovarian cancer cells derived from ascites, were established to evaluate gene expression and the underlying mechanisms of peritoneal invasion [11,20,28,29,47]. However, to date, the physiological features of ovarian cancer in the ascites and at its distal site of metastasis have yet to be packaged into a model that can be used to evaluate the transport of therapeutic agents and delivery vehicles for ovarian cancer applications.

Previous work has utilized single-cell-type 3D cultures of ovarian-only cancer cells either from patient tissue or from cell lines for purposes such as drug screening [48]. However, it is difficult to establish ovarian cancer cell lines, and there has been effort to standardize the process [39]. Heterotypic ovarian epithelial and fibroblast spheroid cocultures have been formed by isolating ovarian epithelial and fibroblast cells from patient tissue samples; however, these cocultures are used less frequently relative to single-cell-type cultures, due in part to the need to obtain human fibroblasts from patient samples or animals (e.g., mouse) as well as the lack of reproducibility and inability to control cell-to-cell distance [40].

Recently, two studies created spheroid cocultures using established ovarian cancer cell lines with the human MRC-5 lung fibroblast cell line. One effort focused on high-throughput 3D-printing of OVCAR-5 and MRC-5 cells [40], and the other on the evaluation of Dicer reprogramming of MRC-5 cells when cocultured with SKOV-3 cells [41]. MRC-5 cells (i.e., lung fibroblasts) have been cocultured with lung epithelial cells (e.g., [42]) and have also occasionally been cocultured with other cancer cell types, such as pancreatic cells [63]. An advantage of MRC-5 cells is that they have been widely characterized since their inception in 1966, especially for their use in vaccine production [64]. They also seem to be the only human fibroblast cell line so far proven to thrive in ovarian cell cocultures. However, the MRC-5 cell line was originally established from cells obtained from the lungs of a healthy human male whose gestation was aborted at 14 weeks due to the mother having psychiatric issues [45], which presents ethical issues in its origin. Further, unlike regular fibroblasts in ovarian tissue, MRC-5 cells have been found to have stem-cell-like properties [46], which creates a potential disparity when attempting to represent cancer-associated fibroblasts in ovarian cancer. In order to more faithfully mimic the ovarian cancer environment, future work will need to explore assembling the 3D coculture platform presented here with ovarian fibroblasts.

The ovarian spheroid model in this study adopts an established framework used for multicellular spheroid models of other cancer types and further integrates physiological characteristics germane to the ascites and peritoneum of ovarian cancer, that lead to a more invasive and therapeutically resistant phenotype [34,35,36]. Specifically, a 3D heterotypic model composed of ovarian adenocarcinoma and fibroblast cells represents a closer step to recapitulating the mobilized nodule-like micrometastases observed in ascites that have migrated from the primary tumor site to the peritoneum. Embedding these multicellular nodules in an ECM mimetic is designed to represent the physiological junction where micrometastases reach and invade the coelomic wall [11,20,49]. We acknowledge that although SKOV-3 and MRC-5 are compatible cell lines and representative of EOC behavior in vivo, the distinct heterogeneity of ovarian cancer is difficult to replicate. Future studies will continue to build upon this coculture foundation, to develop a platform that more closely resembles the composition, morphology, expression, and motility of cancer cells observed in patients.

In addition to the complexity offered by heterotypic cocultures, the constituents of the TME play a significant role in tumor progression. PMX is an ECM mimetic that has been successfully used to promote tumor-like features in other spheroid types. The ability of PMX to promote the growth and migration of MRC-5 and SKOV-3 cells made it a suitable choice with which to develop this model. Furthermore, the composition of PMX can be tightly controlled, improving accuracy and reproducibility of results, unlike other natural hydrogels such as Matrigel that may have batch-to-batch variability [16]. Lastly, PMX has been shown to be highly responsive to tumorigenic microenvironments and to reorganize into an architecture that is refractory to treatment, while promoting cell invasion [50]. Therefore, we hypothesized that implementing these features into a 3D cocultured ovarian spheroid model would result in a more representative system in which to evaluate the transport of new and existing therapeutics and delivery vehicles.

In previous work, we studied the impact a 3D tumor microenvironment formed from a single-cell type, via hanging drop or liquid overlay model, had on NP transport [31,32,33]. Relative to traditional late-stage EOC therapy, which combines cytoreductive surgery with platinum-based chemotherapy in a mostly nonselective and highly toxic approach, more biocompatible and targeted approaches are being sought after to treat late stage EOC [65,66,67]. Nevertheless, despite preclinical successes, the clinical application of ovarian cancer delivery platforms is still limited by the molecularly diverse nature and heterogeneity of the ovarian cancer microenvironment [68]. While a multitude of targeted approaches are being developed, limitations in immunotherapy, gene, and drug delivery continue to be attributed to a lack of understanding of the microenvironment-delivery vehicle. These observations further highlight the need for representative models in which to study these properties.

Previous work in our group evaluated the impact of surface modification on NP penetration within a single-cell type, hanging drop, and liquid overlay spheroids. While a better understanding of NP properties that facilitate transport and cell internalization at the tumor periphery and bulk was obtained from these studies, it has been acknowledged that more complex spheroids, which integrate multiple cell types in different tumor microenvironment conditions (e.g., ECM, cell activation, and hypoxia) may provide a more accurate picture of the clinical challenges facing NP delivery. For example, to more accurately predict NP distribution in vivo, transport limitations posed by the interstitial fluid and subsequent diffusion barriers resulting from CAF remodeling, the ECM architecture, and the induction of hypoxic and acidic regions must be overcome to elicit therapeutic effect [62,69,70].

Given these challenges, the goal of this study was to investigate how multicellular ovarian tumor spheroids, synthesized using the hanging drop method, could be used to model ovarian cancer in various stages of development. First, multicellular (MRC-5 and SKOV-3) spheroids without an ECM mimetic (non-PMX) were cocultured at a 1:1 ratio to resemble the heterotypic morphology of nodules in the peritoneal ascites. Multicellular spheroids activated with TGF-β1 were generally smaller than their nonactivated counterparts, regardless of tissue oxygenation (Figure 2), suggesting the presence of a denser and more aligned ECM. These observations are in agreement with recent work that studied the invasive impact of EMT transition on cancer cells, particularly in the presence of a dense and aligned ECM network that acts as a “highway” for cell migration [71]. Contractile forces generated by CAFs are maintained and reinforced by the deposition of collagen, creating a force imbalance, which eventually results in the stiffening of fibral components [72,73,74,75]. A similar mechanism may explain the observed decrease in spheroid size in the presence of activated fibroblasts. More contractile cells stiffen and align the ECM anisotropically, resulting in a smaller spheroid diameter [72,73,74,75,76]. Importantly, the somewhat decreased sizes observed in activated fibroblast groups in this 3D model compare well with the documented morphological changes seen in ovarian cancer in vivo. The findings of densified microarchitecture seen in Figure 2, in an activated stromal environment, are consistent with the high-density backbone of heterotypic nodules derived from ovarian cancer ascites [11,20,49]. In this set of experiments, cells were cocultured in a 1:1 ratio. Due to different growth profiles, point-to-point variations in collagen and other ECM constituents were not controlled for. In future work, a more in-depth analysis of ECM constituent density via staining or western blot techniques may be used to complement the observations of cell proliferation and growth to account for changes in the presence or absence of nanovector transport.

In addition to evaluating the impact of activation on spheroid growth, non-PMX spheroids were cultured in normoxic and hypoxic conditions to determine the effect of oxygenation on growth. The observed changes in the integrity of multicellular spheroids as a function of hypoxia in Figure 2 are also consistent with the stages of ovarian metastasis—particularly in ascitic fluid [26]. Hypoxic regions within ovarian tumors develop in response to vascular depletion, hemostasis, and tissue diffusion limitations. Studies aimed specifically at evaluating the effects of hypoxia on ovarian cancer cells have shown a clear downregulation of E-cadherin via transcriptional repressor protein SNAIL for both SKOV-3 and OVCAR-3 cells in the presence of hypoxia. This downregulation of E-cadherin is widely believed to alter cellular affinity and promote a more aggressive and invasive phenotype. Several studies have also evaluated the effects of hypoxia on fibroblast cells and their contribution to a pro-tumorigenic microenvironment. MRC-5 cells studied in vitro exposed to hypoxic conditions, showed increased hypoxia-inducible factor (HIF-α), a marker for hypoxia in cells, resulting in a stiffened and aligned ECM that was more invasive for pancreatic cells [77,78]. Although these effects of chronic hypoxia ultimately lead to cell cycle stability and therapeutic resistance, hypoxic damage initially sustained within the harsh environment of the ascites of ovarian cancer causes physiological changes that impact the integrity and morphology of the spheroid, namely apoptosis and reduced cell proliferation [20,25,26,79,80].

Whereas non-PMX spheroids cultured in normoxic conditions increased in size (maximum cross-sectional radii) over 5 days, spheroids cultured in hypoxic conditions demonstrated significant decreases in size (Figure 2). It is understood that in normoxic conditions, the proliferation zone, exposed to a more favorable oxygen environment, continues cell cycle division, while in hypoxic conditions, the proliferation zone undergoes irreversible DNA damage, promoting apoptosis. In Figure 2B, cells appear to demonstrate blebbing membranes and extracellular debris, suggesting hypoxia as a contributor to diminished proliferation and decreased spheroid size. Hypoxic spheroids were more diffuse and irregular in morphology, consistent with previous observations of hypoxic tumor growth in vitro [81,82] and in vivo [83,84,85,86,87,88] and as predicted by in silico modeling [52,89,90,91].

Next, we sought to evaluate the effects of TGF-β1 activation, hypoxia, and PMX incorporation on tumor cells embedded in an ECM mimetic (PMX), seeking to model micrometastatic invasion of the coelomic wall. In this peptide-based scaffold, the most significant changes were observed by comparing PMX spheroid cell migration and growth relative to non-PMX conditions. The changes in tumor size and morphology, observed in non-PMX conditions, were relatively minor compared to radial increases observed in PMX from days 2 to 5. We attribute the more significant changes in migration and spheroid size to the growth-promoting ECM composition derived from PMX. Indeed, relative to the impact of PMX, cell activation and hypoxia seemed to have a lesser impact on spheroid growth. An exception was observed, for nonactivated spheroids in hypoxic conditions, which appeared to attenuate the effect of tumor growth over 5 days, likely due to hypoxia-induced cell quiescence.

Applying this model to evaluate NP penetration, we sought to assess two distinct types of particles used in our previous work with hanging drop models. Cell-penetrating peptides such as MPG have a unique ability to facilitate intracellular uptake via an endosomal mediated pathway [33], whereas the hydrophilic and “stealth” quality of PEG modification (at this molecular weight) is known to inhibit NP adhesion to its surroundings and enhance transport. In our work, we observed distinct differences in NP transport between non-PMX and PMX-embedded spheroids. In the PMX-embedded spheroids, representing distal site invasion (Figure 6 and Figure 7), a significant decrease in NP penetration was observed for both MPG and PEG surface-modified NP groups compared to the ascites stage modeled in Figure 4 and Figure 5. These results highlight the significant impact that ECM density may have on NP penetration, in particular at later stages of tumor progression, potentially due to the ability of PMX to induce reorganization into a more invasive and transport-resistant architecture [50].

The simplified non-PMX model results provide data consistent with our previous studies where MPG NPs accumulated in the spheroid periphery (or at the surface) and PEG NPs distributed more deeply and in greater amounts within the spheroid. Interestingly, MPG NP transport was limited in normoxic conditions, while MPG NPs distributed more readily in hypoxic-cell-activated spheroids. These data suggest that hypoxia may enhance NP distribution due to higher cell death and thus less dense surroundings. Alternatively, in non-PMX spheroids cultured in normoxic/nonactivated conditions, PEG particles demonstrated superior transport.

## 5. Conclusions

Overall, these findings suggest that NP transport is diminished relative to that observed in less complex spheroid models [92,93,94], signifying the relevance in evaluating NPs in multicellular complex environments. While this information highlights the obvious challenges in achieving NP transport, vehicle evaluation in a more complex tumor model may identify stages in which different particle types may be evaluated and potentially improved to more conservatively and realistically estimate delivery to tumor sites. Based on these results, we envision that MPG particles may be effective vehicles for chemotherapeutic delivery prior to mesenchymal invasion, in targeting the CAF backbone of ascitic nodules, to potentially prevent peritoneal implantation [20]. By contrast, the ability of PEG particles to traverse more deeply into the intratumoral environment may be a valuable adjunct, especially in therapeutically resistant microenvironments of metastatic ovarian cancer. Multifunctional particles may also be explored to target different stages of cancer progression. Ultimately, a long-term goal is to enable the evaluation of safety, efficacy, and transport for various tumor-targeted delivery vehicles and determine clinical potential.

## Data Availability

Data will be provided upon request.

## Supplementary Materials

The following are available online at https://www.mdpi.com/article/10.3390/pharmaceutics13111891/s1, Figure S1: Representative average intensity composites of non-PMX spheroids including nonactivated and activated cells cultured in normoxic and hypoxic culture conditions, as a function of NP surface modification. Images depict slices in the z-plane at the midpoint of the z-stack. Green channel represents coumarin 6 NPs, while red represents DAPI stained cells. Scale bars represent 100 μm. Figure S2: Representative average intensity composites of PMX spheroids including nonactivated and activated cells cultured in both normoxic and hypoxic conditions, as a function of NP surface modification. Images depict slices in the z-plane at the midpoint of the z-stack. Green channel represents coumarin 6 NPs, while red represents DAPI stained cells. Scale bars represent 100 μm.
