# Supplementary figures and images for: Multicellular Ovarian Cancer Model for Evaluation of Nanovector Delivery in Ascites and Metastatic Environments
Source: Pharmaceutics. 2021 Nov 8;13(11):1891. doi: 10.3390/pharmaceutics13111891 (PMC8625169; doi:10.3390/pharmaceutics13111891)

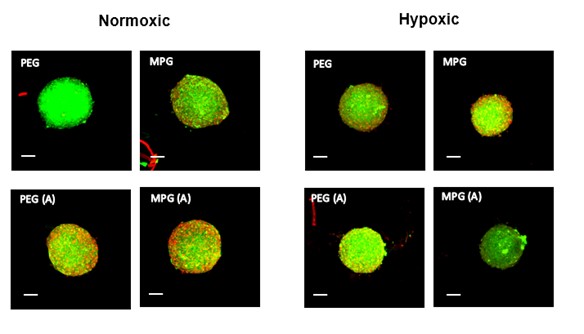

Supplement: Supplementary file 1 [file pharmaceutics-13-01891-s001.zip › Figure_S1.jpg]

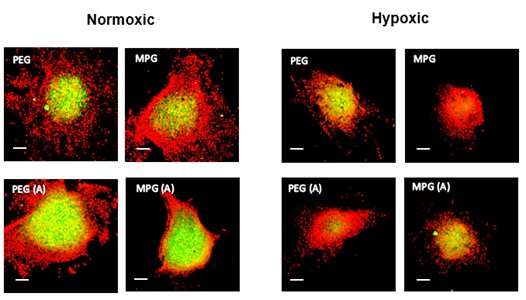

Supplement: Supplementary file 1 [file pharmaceutics-13-01891-s001.zip › Figure_S2.jpg]
